# Flight feather development: its early specialization during embryogenesis

**DOI:** 10.1186/s40851-017-0085-4

**Published:** 2018-01-16

**Authors:** Mao Kondo, Tomoe Sekine, Taku Miyakoshi, Keiichi Kitajima, Shiro Egawa, Ryohei Seki, Gembu Abe, Koji Tamura

**Affiliations:** 1 Department of Developmental Biology and Neurosciences, Graduate School of Life Sciences, Tohoku University, Sendai, 980-8578 Japan; 2 Mammalian Genetics Laboratory, Genetic Strains Research Center, National Institute of Genetics, 1111 Yata, Mishima, Shizuoka, 411-8540 Japan

**Keywords:** Flight feather, Feather development, Chick embryo, Invagination

## Abstract

**Background:**

Flight feathers, a type of feather that is unique to extant/extinct birds and some non-avian dinosaurs, are the most evolutionally advanced type of feather. In general, feather types are formed in the second or later generation of feathers at the first and following molting, and the first molting begins at around two weeks post hatching in chicken. However, it has been stated in some previous reports that the first molting from the natal down feathers to the flight feathers is much earlier than that for other feather types, suggesting that flight feather formation starts as an embryonic event. The aim of this study was to determine the inception of flight feather morphogenesis and to identify embryological processes specific to flight feathers in contrast to those of down feathers.

**Results:**

We found that the second generation of feather that shows a flight feather-type arrangement has already started developing by chick embryonic day 18, deep in the skin of the flight feather-forming region. This was confirmed by *shh* gene expression that shows barb pattern, and the expression pattern revealed that the second generation of feather development in the flight feather-forming region seems to start by embryonic day 14. The first stage at which we detected a specific morphology of the feather bud in the flight feather-forming region was embryonic day 11, when internal invagination of the feather bud starts, while the external morphology of the feather bud is radial down-type.

**Conclusion:**

The morphogenesis for the flight feather, the most advanced type of feather, has been drastically modified from the beginning of feather morphogenesis, suggesting that early modification of the embryonic morphogenetic process may have played a crucial role in the morphological evolution of this key innovation. Co-optation of molecular cues for axial morphogenesis in limb skeletal development may be able to modify morphogenesis of the feather bud, giving rise to flight feather-specific morphogenesis of traits.

## Background

**Fig. 1:**
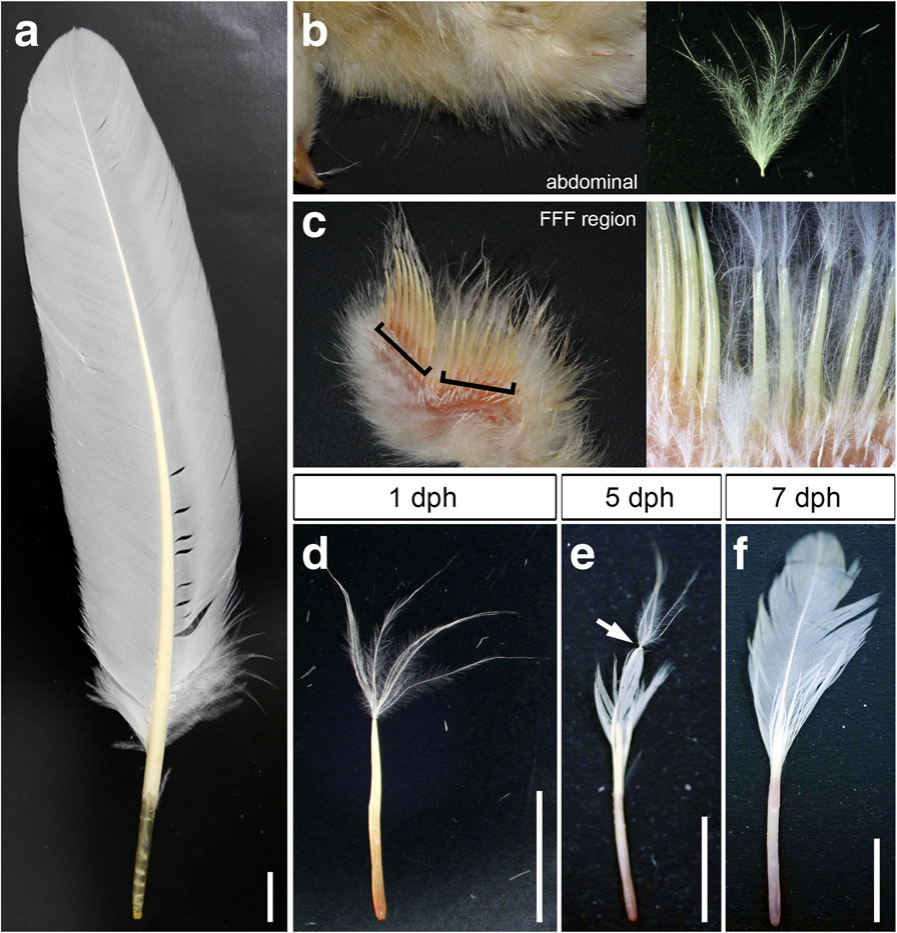
Development of remex-type flight feathers after hatching. **a** A flight feather in the adult chicken. **b** Natal down feather in the abdominal tract at 1 dph. An uprooted abdominal natal down feather is shown on the right side. **c** Ventral view of the FFF region at 1 dph. Some rows of feathers in the ventral surface of the wing are trimmed. Staples indicate the FFF region. An magnified photograph is shown on the right side. **d**–**f** A feather uprooted from the FFF region at 1 dph (**d**), 5 dph (**e**), and 7 dph (**f**). Note that in **e**, a distal downy part is connected with a proximal bilateral part that starts opening (white arrow). Scale bars: 1 cm

Feathers are specialized skin derivatives (integument appendages) that are unique to extant/extinct birds and some non-avian dinosaurs; in living birds, most of the body is covered with some kinds of feathers. Feathers in birds, which serve more than 20 different functions [1], including thermoregulation, physical protection, tactile sensation, various types of visual signaling such as display in courtship, and flight, are functionally refined integument appendages. Bird feathers can be classified into many types, such as down, bristle, and contour feathers (including flight feathers), and the evolution of various feather types has enabled feathers to exert a variety of functions. The flight feather is the most evolutionarily advanced type of feather [2, 3]. Flight feather evolution was one of the keys for bird evolution because powerful flight of birds is made possible by flight feathers, which have asymmetric shapes of the vanes along the rachis [4] (see also Fig. 1) that aero-mechanically enable birds to fly [1]. There are mainly two body regions where flight feathers grow; one region (for remiges) is the posterior margin of the wing (forelimb) and the other (for rectrices) is the lateral margin of the tail. A feather is an exterior structure on the skin in general, but the shaft (quill) of the remex-type flight feather is anchored to the bone (ulna and metacarpal/phalanges of digit 2) with extra muscles and neural networks for motor control [5]. These bones have protrusions on the surface (quill knob) that directly attaches to the proximal end of the flight feather calamus (calamus; the short, tubular base of the quill), which functions as mechanics of flight [6, 7].

During embryogenesis and molting, all feathers develop from primordial feather buds with follicles (feather follicles) at the base of it, and repetition of feather development enables cyclic molting. A chick after hatching appears to be covered with fluffy downy feathers (natal down feathers), and diversification of feather types emerges in the second or later feathers that form at the first and following molting. In the chicken, the first molting generally begins at around two weeks post hatching [4], and it is therefore possible that complex features of the flight feather would be provided at molting after hatching. However, it has been stated in some previous reports that the first molting from the natal down feather to the remex-type flight feathers is much earlier than that for other feather types [4, 8, 9] (see also Fig. 1b-f). Moreover, Hosker [8] reported that the feather follicles for the remiges reach the wing bone by the time of hatching, although details remain obscure. In order to clarify the early origin of flight feathers, we speculated that flight feather development may start much earlier as an embryonic event in mid-development in chicken. If this is the case, it is possible that the mechanism for morphogenesis of evolutionally advanced features of the flight feather has not simply added to the mechanism for the morphogenesis of the primitive types of feathers but is instead a modified/peculiar morphogenetic process.

Considering the evolutionary and functional peculiarities of the flight feather, when and how flight feathers develop are important issues for feather biology in general. The aim of this study was to determine the incipient morphogenesis of the flight feather, focusing on when morphological traits of the remex-type feather start developing and how special morphologies of remiges (e.g., attachment between the flight feather calamus and the bone) are established. There are already many descriptions of the morphology, morphogenesis and molecular characters of the flight feather. Using such information, we present here morphological, histological, and molecular marker observations of the flight feather bud, and describe its developmental sequence. Integrating such descriptive information will also serve as the basis of feather diversification. Our results suggest that flight feather morphogenesis in the chicken is an embryonic event starting from the middle of chick development and that the first down feathers in the flight feather-forming region, the follicles of which have already reached the bone, are special feathers that are different from other natal down feathers.

## Methods

### Animals

Chicken eggs (*Gallus gallus*) were purchased from a local chicken ranch (Iwaya poultry farm) and incubated at 38 °C, and embryos were staged according to Hamburger and Hamilton (1951) [10] (HHstage). White Leghorn chicks and adult chickens after hatching were supplied by the National BioResource Project (NBRP) Chicken/Quail of the MEXT, Japan, and the age of the chicks was ascertained by the NBRP supplier.

### Histology

#### Hematoxylin and eosin (HE) staining

Forelimb buds and a square area of abdominal skin, including feather follicles, from staged chicken embryos were used for histology. Samples for HE staining were fixed with Bouin’s fixative (9% formaldehyde, 5% acetic acid and 75% saturated picric acid) at room temperature (RT) overnight with shaking. The samples were washed with 70% ethanol (with saturated lithium carbonate) for 5–7 days followed by dehydrating with 80%, 90% and 100% ethanol (twice for 100%). Then the samples were permeated with xylene at RT once, 1:1 xylene/paraffin (paraplast, Leica) at 45 °C once, and paraffin at 60 °C three times and embedded in paraffin at RT. The paraffin blocks were sectioned at 10 μm in thickness on slide glasses (MAS-coated, MATSUNAMI). The sectioned samples were immersed in xylene for 20 min and then quickly immersed in 100% (twice), 90% and 70% ethanol and then rinsed in water. The sections were stained with hematoxylin solution (Muto Pure Chemicals) at RT for 1 min and washed with water followed by eosin staining for 30 s and water washing. The samples were dehydrated with a series of ethanol (70%, 90% and 100% ethanol) and xylene and were mounted with Eukitt (ASONE).

#### Elastica van Gieson (EVG) and Alcian blue (AB) staining

The method for EVG + AB staining was essentially the same as that for HE staining except for the thickness of a section (15 μm). See Hayashi et al. [11] for details of EVG + AB staining. We made some modifications for staining: coloring times for Weigert’s hematoxylin solution, alcian blue solution and van Gieson’s solution were for 3 min, 10 min and 3 min, respectively, in this study.

### In situ hybridization

Forelimb buds and a square of abdominal skin with hypodermal tissues were excised from staged chicken embryos and processed for whole-mount in situ hybridization as described previously [12] using antisense RNA probes for chick *shh* (see [12, 13] for detailed information of *shh* RNA probes). In the later stages of feather morphogenesis, *shh* is expressed in the marginal plate epithelium that will undergo cell death, providing spaces between the barbs [14–16].

In situ hybridization of frozen sections was performed essentially by the same method as that described by Yoshida et al. [17] using antisense RNA probes for chick *shh*.

## Results

### Flight feather morphology and development after hatching

In all experiments described below, we used flight feathers in the zeugopod for specimens of flight feathers (Fig. 1a) and their primordia, and compared them with abdominal feathers (Fig. 1b). In this study, we defined the place where remex-type flight feathers (remiges) are formed as the “flight feather-forming region (FFF region, see Fig. 1c)”, which is the posterior margin of the zeugopod and autopod in the wing. In the FFF region, the first generation of feathers is down-type and the second generation is remex-type. An old textbook (Figs. 138–141 in [4]) showed bar charts representing the history of feathers on several body tracts of the chicken, in which the first molting in the FFF region was shown to be very early in the chick, but no definite data were shown in that report. An old report by Hosker [8] showed a feather from the wing of a one-day-old chick (Fig. 27 in [8]) that has clear two parts arranged linearly, but there is no mention about whether that specimen is a flight feather in the FFF region in that report. Therefore, we decided to repeat these observations and confirm how early the first molting occurs in the FFF region in the chicken. In contrast to the morphology of natal down feathers on the abdominal surface at one day post hatching (dph) (Fig. 1b), feathers in the FFF region are composed of two different parts: a distal part resembling down feathers and a proximal part of a shaft without barbs visible at 1 dph (Fig. 1c, d) as described by Hosker [8]. Developing barbs here seem to be still inside the sheath of this proximal part of the developing feather and are therefore not visible yet as described by Alibardi [18]. The root of the proximal part is deeply embedded in the wing. At 5 dph, the distal portion of the proximal part begins to open, and barbs with a bilateral shape along the rachis can be seen (Fig. 1e, showing that the down feather is still on the distal end of the opening bilateral barbs). The remex-type asymmetric barbs further open at 7 dph, and the distal down part has dropped from this specimen (Fig. 1f). A series of observations (Fig. 1) confirmed that the distal part at 1 dph is the first down feather that connects with the second remex-type feather, although the remiges have not been opened yet at that time (Fig. 1d), suggesting that the first molting starts at around the hatching stage. It seems that the remex-type feather has already started developing at embryonic stages before hatching.

### Flight feather morphology and development before hatching

**Fig. 2:**
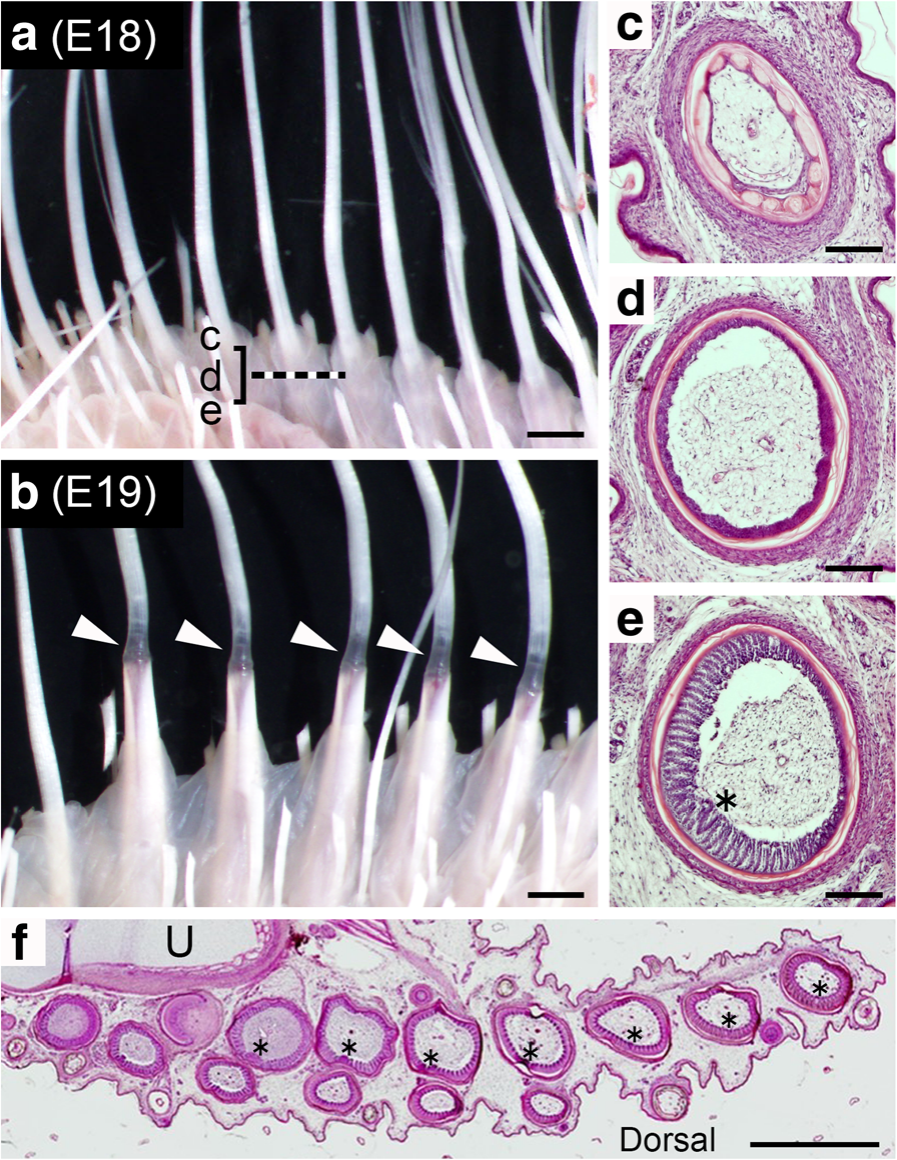
Development of remex-type flight feathers before hatching. **a, b** Ventral view of the FFF region at E18 (**a**) and E19 (**b**). Scale bars: 1 mm. Arrowheads indicate joints between distal and proximal parts of the elongated feather bud in the FFF region. **c**–**e**, Sections of the feather bud under the skin at E 18; (**c**), a shallow level, (**d**), an intermediate level and (**e**), a deep level. Scale bars: 100 μm. Asterisk in (**e**) indicates the position of the prospective rachis that exhibits an irregular pattern of barb ridges. **f** Rows of feather buds in the posterior wing, including the FFF region, at E18. Distal to the right and dorsal to the bottom. U: ulna. Asterisks indicate the position of prospective rachis, showing that the position is biased to the dorsal. Scale bar: 100 μm

In the FFF region at embryonic day 18 (E18), we observed a row of epidermal sheath for the elongated feather bud, and there was no visible connection of the two feather primordia as judged by externals (Fig. 2a). In contrast, at E19, the epidermal sheath in the FFF region showed a clear joint (white arrowheads in Fig. 2b) that resembles a connection of columns between the second mature remex and third immature remex at the second molting in the adult chicken [4], suggesting that the proximal portion to the joint is the second generation of feather (remex-type). The second feather may thus have already developed before E19. We expected that there may be a primordial remex-type feather under the skin at E18, and histological observations of sections (indicated by a dashed line in Fig. 2a) were attempted. At a shallow level (Fig. 2c), radially symmetric barb primordia on the circumference could be seen in the feather follicle, and this configuration resembles that of a primordial natal down feather in which all barb ridges are parallel to the long axis [4, 19]. At the middle level (Fig. 2d), there was a layer where no barb primordia were visible. At a deeper level (Fig. 2e) of the same feather follicle as that shown in Fig. 2c and d, there was a bilateral barb arrangement on the circumference, and this configuration indicates a typical flight feather primordium [20]. These observations revealed that the second generation of feather that shows a remex-type feather arrangement has already started developing by E18 deeply in the skin of the FFF region. In addition, the position of the rachis primordium in each feather bud (indicated by asterisks in Fig. 2f) was biased to the dorsal side of the wing.

### Second generation of feather development in the FFF region

**Fig. 3:**
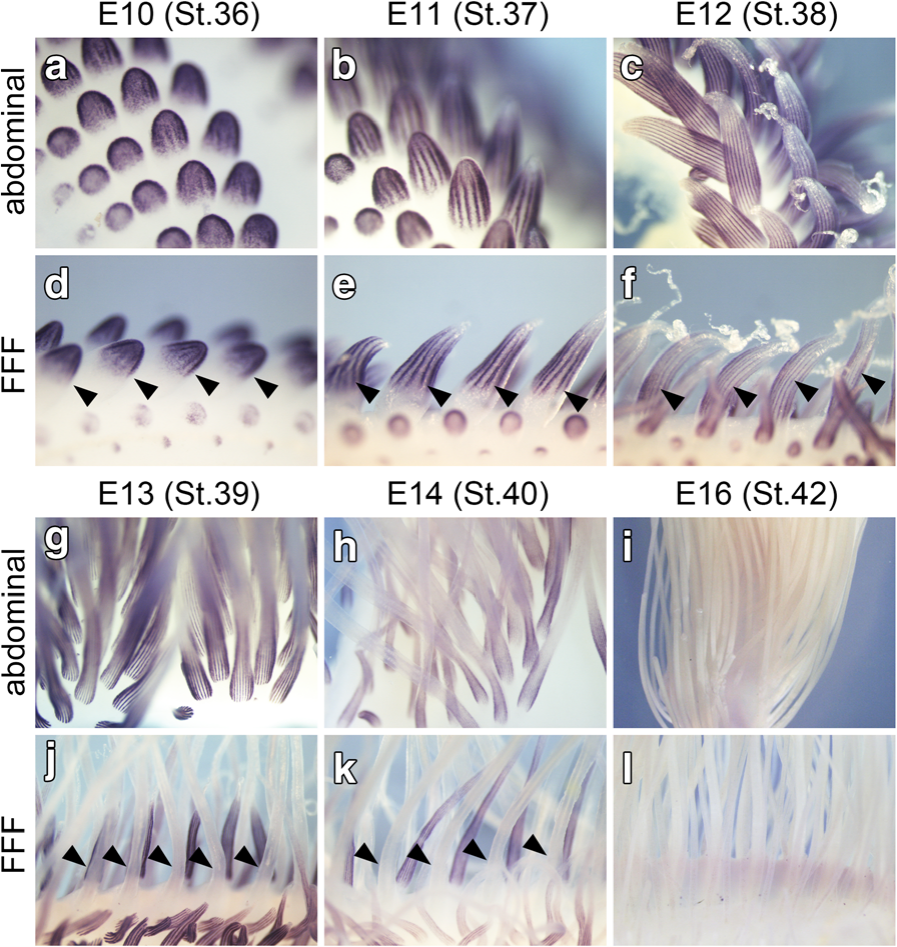
Whole-mount observations of *shh* expression in the abdominal and FFF regions. **a**–**c**, **g**–**i** *shh* expression in the abdominal tract by whole-mount in situ hybridization from E10 (HHstage 36) to E16 (HHstage 42). **d**–**f**, **j**–**l** *shh* expression in the FFF region. Arrowheads in (**d**–**f**), (**j**), (**k**) indicate feather buds in the FFF region: note that in **j** and **k**, feather buds in the FFF region have little *shh* expression

To further investigate when the remex-type feather development starts, we analyzed the expression pattern of a gene, *sonic hedgehog* (*shh*). Expression of *shh* starts early in the feather placode and then remains in the epidermal marginal plate (presumptive inter-barb region) of the feather bud [14–16], and we therefore thought that we could estimate feather types by the *shh* expression pattern. At E10, many feather buds protuberated on the abdominal surface of the body trunk (Fig. 3a), and *shh* was expressed in the epidermis of the protrusion, with the pattern of expression becoming obvious at E11 (Fig. 3b) and E12 (Fig. 3c). In the abdominal feather buds, *shh* expression showed a radially symmetric pattern that corresponds to the barb arrangement of a natal down feather. In the FFF region, a line of feather buds (arrowheads in Fig. 3d–3f, 3j, 3k) were much larger than those in the surrounding area of the wing skin surface. Expression of *shh* in feather buds in the FFF region showed a radially symmetric pattern similar to that in abdominal feather buds. We never observed a bilateral pattern of *shh* expression (indicating the pattern for contour feathers) by whole-mount in situ hybridization on the embryonic days we conducted observations. Thus, it appears that the elongating feather bud in the FFF region gives rise to down feather during this developmental period of E10-E12. The expression of *shh* in the abdominal feather buds continues to E13–15 (Fig. 3g, h and not shown), and signals for *shh* expression could not be detected at E16 (Fig. 3i). On the other hand, *shh* expression in the FFF region had disappeared by E13 (black arrowheads in Fig. 3j), although in surrounding feather buds *shh* continued to be expressed (Fig. 3j and k) and the *shh* expression disappeared by E16 (Fig. 3l), indicating that the first generation of feather development in the FFF region ceases much earlier than that in other feather buds. Thus, feather development of the first generation in the FFF region may proceed more rapidly. We found such heterochronic modification of *shh* expression, but did not detect a second generation of remex-type feather formation by whole-mount observation of the *shh* expression pattern.

**Fig. 4:**
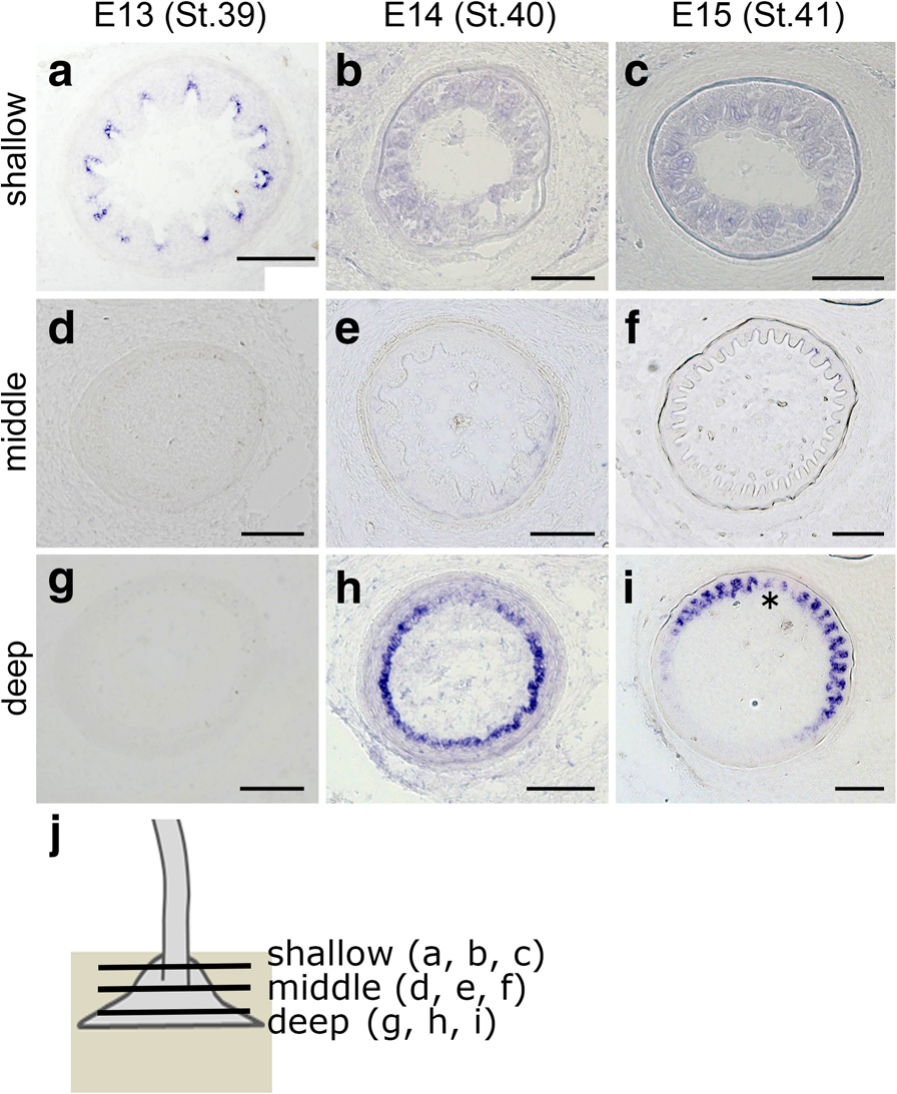
*Shh* expression detected by section in situ hybridization in the FFF region. Cross sections around the bottom region of feather buds in the FFF region at E13 (**a**, **d** and **g**), E14 (**b**, **e** and **h**) and E15 (**c**, **f** and **i**) at shallow (**a**–**c**), middle (**d**–**f**) and deep (**g**–**i**) levels under the skin. Asterisk in **i** indicates an irregular pattern of *shh* expression that represents the position of the prospective rachis. **j**. an illustrated image showing positional relation of sections (**a**–**i**) along depth direction. Scale bars: 100 μm

As can be seen in histological images shown in Fig. 2, it remained possible that the second generation of feathers was developing in a deep area under the skin, and we thus examined *shh* expression by in situ hybridization for sections of feather buds in the FFF region (Fig. 4). At E13, when a natal feather pattern could not be detected by whole-mount observation (Fig. 3j), clear expression of *shh* was detected by section in situ hybridization at a relatively shallow level under the skin (Fig. 4a, see Fig. 4j, an illustrated image showing positional relation of sections along depth direction). The expression of *shh* was circumferentially spotted with a radially symmetric pattern, typical for a natal down feather [21]. The feather development generally proceeds in a proximal-to-distal direction, and the more distal region of a feather bud develops to be more mature. This could account for the continued expression of *shh* in the proximal region of the feather bud retains (Fig. 4a), while *shh* expression ceases in the distal external region (Fig. 3j). Faint expression of *shh* at a shallow level was detected until E15 (Fig. 4b and c). At a middle level, clear *shh* expression was not detected in the period we examined (E13-E15, Fig. 4d, e and f). Interestingly, at a deep level of the feather bud in the FFF region (slightly upper from the bottom), we detected a clear signal of *shh* at E14 and later (compare Fig. 4g and Fig. 4h, i). The expression pattern of *shh* at this depth became not radially symmetric but bilateral (Fig. 4i, a prospective rachis is indicated by an asterisk), resembling the barb arrangement in the feather follicle of the molting flight feather in the adult [20]. We found that the second generation of feather development in the FFF region, which gives rise to remex-type feathers, appears to start by E14 at latest, a week before hatching.

### A unique morphogenetic process for first generation of feathers in the FFF region

**Fig. 5:**
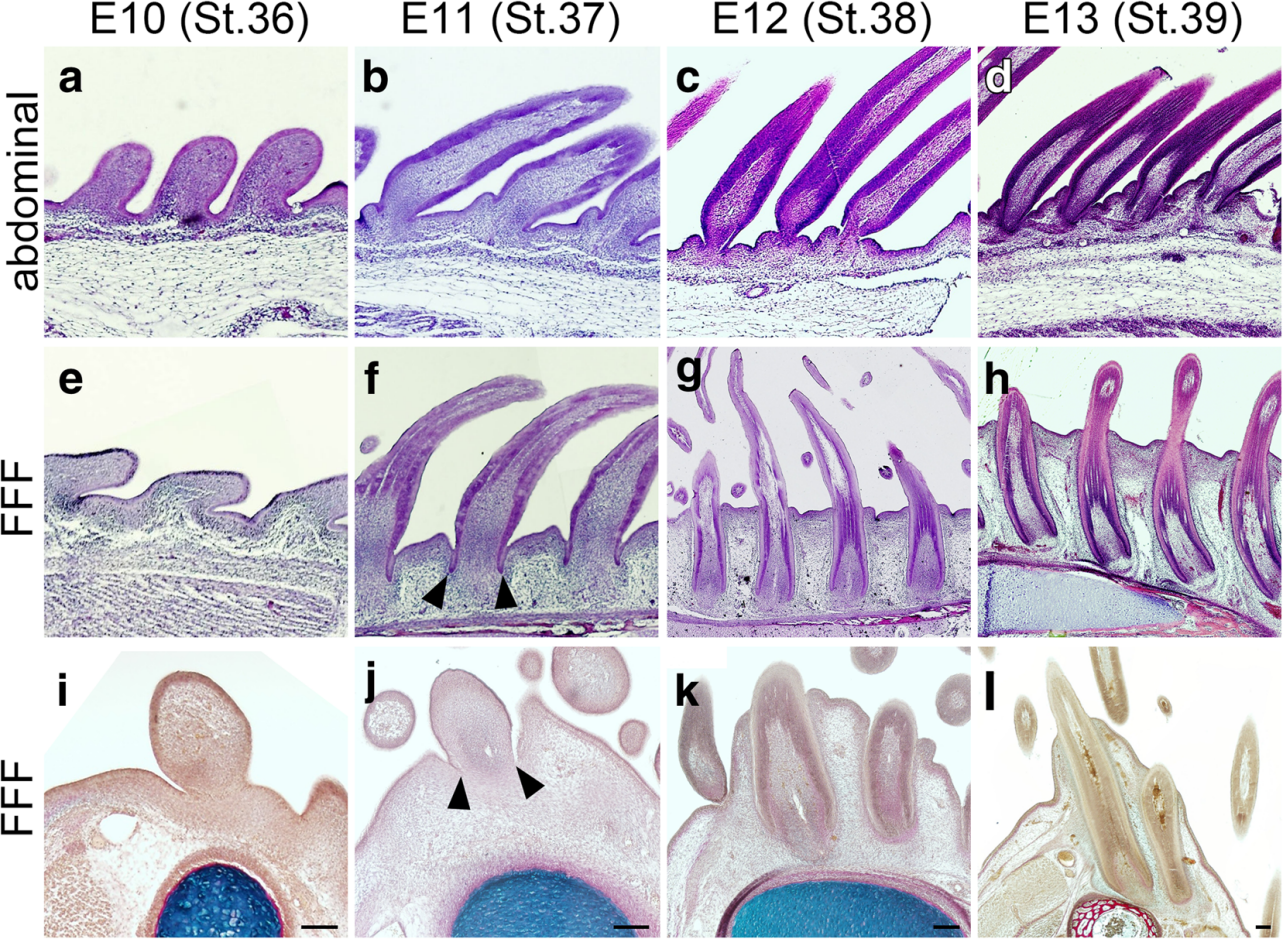
Deep invagination of the feather bud toward the bone primordium in the FFF region. **a**–**h** Sagittal sections of abdominal skin (**a**–**d**) and coronal sections of the FFF region (**e**–**h**) at E10 (**a**, **e**), E11 (**b**, **f**), E12 (**c**, **g**) and E13 (**d**, **h**). Arrowheads in (**f**) indicate the follicular cavity that starts invagination. **i**–**l**. Transverse sections of the FFF region showing the invagination process of the feather bud in the FFF region at E10 (**i**), E11 (**j**), E12 (**k**) and E13 (**l**). Scale bars: 100 μm. The blue core is the cartilaginous rudiment of the ulna, and the red layer surrounding the ulna cartilage is the ossifying bone collar

In the above-described observations of section in situ hybridization, we realized that feather buds in the FFF region extend the base of the calamus in a deep layer under the skin of the wing. This surprised us, as feather buds are generally external structures on the skin surface (Fig. 5d) (see also panels with histological sections of developing chick skin in [16, 22]) and drove us to further observe histologically the process of feather bud formation in the FFF region. Feather buds in the abdominal region develop on the skin surface with underlying mesenchyme (Fig. 5a). The buds grow toward the outside and make a sheath-like structure (Fig. 5b and c). The sheath-like elongated feather bud was a surface structure (Fig. 5d). Although the base of the epidermal sheet made a slight invagination to create a cavity-like bend (follicular cavity, see [3]) and the surrounding dermal layer became thicker, embedding the follicle in the dermis, the feather follicles did not enter the hypodermal layer but remained external to it. On the other hand, in the FFF region, the feather bud showed a peculiar process of development different from that of the abdominal feather bud. At E10 (Fig. 5e), the feather bud in the FFF region looks similar to the abdominal feather bud. External morphology of the feather bud in the FFF region still looks similar at E11 (Fig. 5f, compare to 5b and see also Fig. 4b and e), but there was a slit-like bend of the epithelium surrounding the base of the protruding feather bud (arrowheads in Fig. 5f and j). At E12, the epithelial bend became deeper, and the feather bud created cylindrical invagination into the hypodermal layer (Fig. 5g and k). The proximal end of the feather bud sank into the deep limb mesenchyme and reached the bone (ulna) by E13 (Fig. 5h and l). Observation of a series of transverse sections of the developing wing with bone (in red) and cartilage (in blue) (Fig. 5i–5l) revealed that the base of the feather bud reaches the ulna before ossification of the bone collar is completely finished (Fig. 5k and l, showing the ossifying bone collar in red). We never observed such invagination of the feather bud in the abdominal region (Fig. 5a–5d). These observations strongly suggest that the initial feather bud in the FFF region has a unique morphogenetic process for flight feather-specific traits, deep invagination and direct attachment to the bone, although the first generation of feathers in the FFF region shows an external down feather-like morphology, as seen in Fig. 3.

## Discussion

**Fig. 6:**
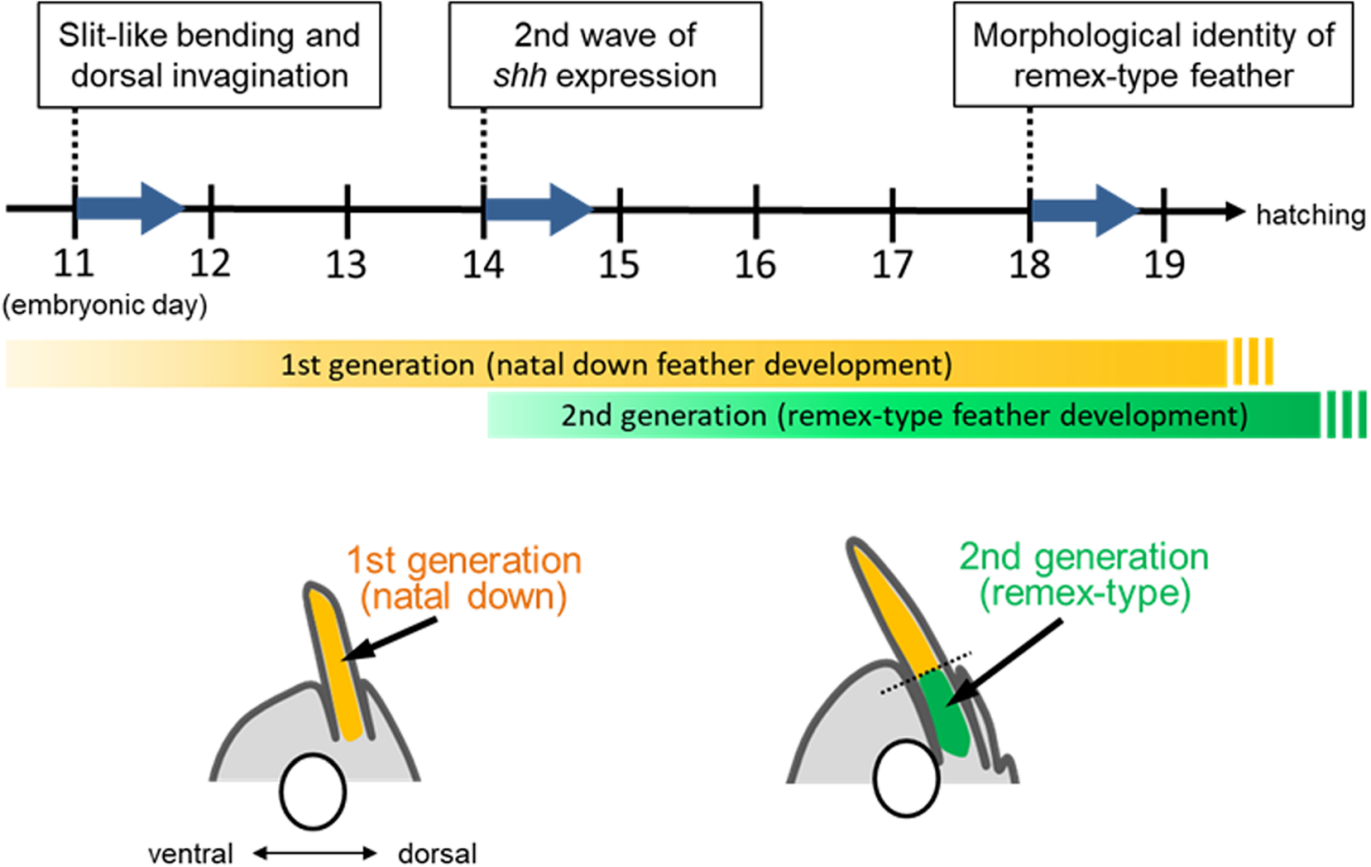
Schematic representation of the developmental process of feather formation in the FFF region. Illustrated images in the lower column indicate posterior halves of transverse sections of the developing wing buds with a feather bud in the FFF region and ulna (ellipse). See the text for details

In this study, we traced the morphogenesis of the flight feather back until embryonic stages and revealed some specialties of its process. In Fig. 6, the morphogenesis from initiation of feather bud formation to later embryogenesis is shown.

The first stage at which we detected a specific morphology of the feather bud in the FFF region was E11, when a slit-like bend forms around the bud. At this stage, the feather bud starts to change its relative position to the skin surface and seems to make invagination into the skin. It is possible that both invagination of the bud and swelling of the surrounding skin occur simultaneously. The invagination continues, and the feather follicle reaches the bone at E13. At this time point, the second wave of *shh* expression (for remex-type) has not yet been initiated, while *shh* is expressed in the external region of the feather bud with a radially symmetric pattern. The radial *shh* expression results in the natal down feather morphology with a radial barb pattern. The second wave of *shh* expression, which has a bilateral pattern, can be detected around the bottom of the feather bud deeply in the wing at E14. Therefore, the feather bud in the FFF region at this stage can be divided into several parts from proximal to distal: *shh*-positive (bilateral), *shh*-negative (as a joint), and *shh*-positive (radial). The proximal bilateral pattern of *shh* expression results in the remex-type feather pattern that is visible externally at around five days post hatching. The remex-type feather with a joint to the first generation of the down-type feather can be seen internally at E18 and externally at E19.

### Invagination of the feather bud in the FFF region and formation of the quill knob

Invagination is a morphogenetic process of epithelial tissue that changes a flat epithelial sheet to complex structures such as tubes, furrows, and capsules. The skin epithelium creates many kinds of structures, such as secretory glands, nails/claws/hoofs, and scales/feathers/hairs, by interacting with the underlying mesenchyme [2, 9, 14, 23, 24]. Morphogenesis of skin derivatives is preceded by epithelial invagination. In the developing feather bud, invagination gives rise to a collar-like bend, the bottom of which serves as a feather follicle, the cellular source of the feather. The feather follicle reaches the dermal tissue, but the invagination is generally not so deep, retaining the follicle in the hypodermal layers of the skin. In contrast, the feather follicle in the FFF region deeply sinks to the posterior-dorsal surface of the bone. Although the mechanism of epithelial invagination of a feather bud in the FFF region is largely unknown, an interesting report by Lin et al. [13] suggested that tubular invagination is achieved through loosening of the bud mesenchyme. Epithelial cell proliferation and elongation in the bud may lead to the formation of a sprout that invades the surrounding loosened mesenchyme, and epithelial-mesenchymal interaction may contribute to the epithelium organization as seen in the morphogenesis of other types of epithelial tissues such as mammary glands [25]. Rows of feather buds more dorsal to the FFF region, which give rise to wing coverts, also make deep invagination into the wing bud mesenchyme (Fig. 5l). Since the feather buds at the ventral side of the wing do not show such deep invagination (Fig. 5k, and not shown), the dorsal property of the limb that is provided by dorsal determinants such as *lmx1* might participate in the deep invagination.

It could be hypothesized that this contact results in the formation of a bump on the bone surface. The quill knob is a trait associated with the flight feather and plays important roles in flight because the attachment point between the knob and the calamus functions as a mechanical link that transfers aerodynamic forces generated by flight feathers to the wing skeleton [5]. The characteristics of the flight feather start developing in the initial process of feather bud formation, earlier than the remex-type feather morphogenesis. The remex-type morphology is thought to be the most evolutionarily and developmentally advanced phenotype of the feather [2, 3] with secondary modification of the developmental process of the feather pattern, but it does not necessarily mean that the modification was added subsequently to morphogenesis of primitive type. Our results suggest that at least some characteristics of the flight feather are provided by modification of processes in its early development.

### Remex-type feather formation

Our observation of *shh* expression in the feather bud of the FFF region revealed that its expression in the FFF region has a non-radial but bilateral pattern at E14 and E15. This suggests that there is the second generation of feather formation, the morphology of which is different from that of natal down in the first generation, already at the embryonic stage before hatching in the FFF region. The molecular mechanism for transition of *shh* expression from radial to bilateral remains unknown. Gradient signaling such as Wnt3a [26] and retinoic acid (RA) [27] are thought to provide the bilateral and asymmetric shape of the feather with a rachis since modification of Wnt3a and RA gradients in the feather bud transforms feather morphology. In these experiments, young adult chickens at the molting stage were focused on, but also in the embryonic stage, change in these signaling pathways from a homogenous pattern to a localized pattern in the feather bud might occur before establishment of bilateral *shh* expression at E14.

Vanes in remiges are bilaterally asymmetric to the rachis, and this asymmetry is more distinguishable in the distal wing (primary remiges). Feo and Prum [28] examined the developmental process of the asymmetric barb shape of rectrices in the tail of adult parrots, and they found that the developing feather germ of rectrices has asymmetry of barb ridges to the rachis in a cross section. We did not detect clear asymmetry of the feather bud in the FFF region, but the *shh*-expressing region shown in Fig. 4i seems to be asymmetric to the hinge of expression (a prospective rachis, indicated by an asterisk), corresponding to the discussion of the origin of asymmetric feathers [18, 29]. It would be interesting to investigate the relationship between asymmetry of the feather pattern and axis formation of the limb. The feather bud in the FFF region and its invagination are biased to the dorsal side of the limb, and the position of the future rachis (asterisk in Fig. 4i) is also biased to the dorsal side of the limb (Fig. 2f). The axis formation of the limb regarding dorsal-ventral, proximal-distal and anterior-posterior may play a role in the axis formation of the feather. We would like to discuss this point further in the next section.

### Hypotheses on specification of the FFF region

The FFF region can be defined as a region satisfying four criteria of topological identity in the bird body. The first criterion is “the forelimb”. The second one is “the zeugopod and autopod”. The third one is “the posterior margin”. The last criterion is “the dorsal side”. The position where the above four criteria meet, “the dorsal side of the posterior margin in the zeugopod and autopod of the forelimb”, is the position for the FFF region. In the process of limb development, each of the four criteria can be specified by specific gene expression patterns and functions, and it is possible that these molecular networks for specifying the four criteria in the limb bud may also be applied to specifying the FFF region and providing peculiar morphogenesis of the feather follicle in the FFF region. We hypothesize that feather formation in the FFF region is special from the beginning, and this enables us to discuss relationships between molecular mechanisms for establishing the above four criteria and feather formation in the FFF region.

#### Criterion 1 for the FFF region: forelimb

The remex-type flight feather in the extant bird is forelimb-specific, although some modern birds such as some species of raptors bear feathers in the hindlimb zeugopod that seem to be important in flight when catching and carrying prey [30]. A reliable marker for the tetrapod forelimb bud is *tbx5*, which has essential functions in initiation of development and morphology of the forelimb [31, 32]. *tbx5* deficiency results in a limbless phenotype, and evidence for the direct function of *tbx5* in flight feather morphology is therefore sparse. Nevertheless, recent findings on avian genetics and embryology have revealed crucial functions of *tbx5* in flight feather formation. There are some mutants (strains) of domestic chickens and pigeons that have flight feather-like asymmetrical feathers in the hindlimb. Domyan et al. [33] found that this intriguing phenotype is associated with *cis*-regulatory changes in the *tbx5* locus that give rise to ectopic expression of *tbx5* in the hindlimb bud, suggesting a role of this gene in flight feather formation.

#### Criterion 2 for the FFF region: zeugopod and autopod

Mayerson and Fallon [34] suggested that feathers arise sequentially and independently within different feather tracts (reviewed in [14]). Johansson and Headon [35] suggested that positional information mediated by transcription factors, including Hox genes, can code regionalization of a skin appendage. Indeed, some homeobox genes show different expression patterns in skin tracts in the chick embryonic body [36–38]. Hox genes are also involved in regionalization of the limb along the proximo-distal axis (from stylopod through zeugopod to autopod); *hoxa11* is a key molecule for zeugopod specification, and *hoxa13* is a key molecule for the autopod, and both of molecules have essential functions in morphogenesis of the regionalization [39–41] (reviewed in [42, 43]), although the direct functions of these Hox genes in flight feather formation remain undetermined. Functional studies should be carried out to determine their role in the establishment of regional differences of feather type. Li et al. [27] reported that retinoic acid signaling mediates a continuum of asymmetric vanes of flight feathers along the proximo-distal axis of the wing, and they discussed the possibility that the limb RA gradient that is used for the proximo-distal limb patterning is somehow imprinted within remex pulp cells and that the limb RA gradient is also used to establish asymmetry levels in remiges along the wing. It is possible that information of the proximo-distal axis in the wing mediated by retinoic acid signaling is embedded into the developing feather bud at the zeugopod and autopod through different expression patterns of Hox genes.

#### Criterion 3 for the FFF region: posterior margin

Flight feather buds appear at the posterior margin of the forelimb bud. The posterior margin is the region where the feather primordia develop earliest in the chick forelimb bud [34]. The posterior margin of the limb bud includes a special mesenchymal region, so-called zone of polarizing activity (ZPA). Anterior implantation of ZPA tissue induces a set of digits with mirror-image duplication, in which large feather buds develop at the anterior margin (with newly-acquired posterior identity) of the duplicated limb [44], suggesting that the zone contributes to the FFF region. Shh, a secretory molecule responsible for polarizing activity of the zone, also induces posterior feather buds when applied to the anterior margin [44]. Furthermore, ectopic flight feathers in pigeon and chicken mutants are biased to the posterior foot, and ectopic expression of *tbx5* in the hindlimb in these mutants is also posterior-biased [33, 45]. Taken together with the fact that flight feathers in the raptor hindlimbs are also posterior-biased [30], it is thought that molecular networks for forelimb identity and posterior identity of the limb morphology coordinate formation of the FFF region. Since *shh* expression in the forelimb ZPA disappears at around HHstage 29 [46] before the feather bud-initiating stage, *shh*-experienced posterior mesenchyme (descendants of mesenchymal cells that had expressed *shh* gene in the early stages) might contribute to the induction of the flight feather bud in the FFF region.

#### Criterion 4 for the FFF region: dorsal side

Quill knobs are formed on the dorsal surface of the bone [7]. This would be because invagination of the FFF feather bud proceeds to the dorsal side (see Fig. 5l), and the proximal end of the feather bud reaches to the dorsal surface of the bone. It is known that dorsal-ventral reorientation of developing chick limb ectoderm makes a bi-dorsal feather pattern, including a double row of flight feather buds [47]. It is also noteworthy that limbless mutants of the chicken, which have no dorsal-ventral boundary due to the complete dorsalization of the early limb field, resulting in no outgrowth of the limb bud, do not form wing (thus no flight feathers) [48]. Interestingly, when the mutant phenotype was partially rescued by application of FGF, double lines of feathers that looked like flight feathers in the sheath appeared at the posterior margin of the developed forelimb [48]. These results strongly suggest a correlation between the dorsal-ventral organization of the limb and flight feather formation, although the molecular mechanism remains unknown. *lmx1*, a determinant of limb dorsal morphology, is expressed in the dorsal part of the developing wing bud [46], and feather placodes in the FFF region are included in the *lmx1*-positive dorsal territory (unpublished observations by TS, TM, KK and KT). However, miss-expressed *lmx1* does not change the ventral feather pattern when it is ectopically expressed in the ventral side of limb bud [46], suggesting that the molecule is not sufficient to provide the FFF region.

The FFF region that satisfies the above four criteria may develop special traits of the feather bud from the beginning of feather development, and one of the first visible specialties in feather development in the FFF region is the formation of a slit-like bend surrounding the feather bud at E11, followed by deep invagination. We recently reported that the gene *Sim1* is expressed in the posterior margin of the zeugopod and autopod in the developing wing of the avian embryo, nicely corresponding to the FFF region [45]. *Sim1* expression that is specific to the region where the above four criteria meet starts at around E7 and ceases at around E14 ([45] and our unpublished observations). Although investigation of the functions of *Sim1* and the other molecules described above in the feather formation are necessary, we suggest that *Sim1* expression in the FFF region is not necessarily too early to contribute to flight feather formation.

## Conclusion

Our results indicated that the second generation for the remex-type flight feather has already started developing before hatching as an embryonic event. The morphogenesis in the FFF region is modified from the beginning of feather morphogenesis as the first generation of the feather also has a unique morphogenetic process.

The flight feather is thought to be the most evolutionarily and developmentally advanced type of feather, and our results presented here suggest that the morphogenesis for the advanced type has been drastically modified from the beginning of feather morphogenesis. At the beginning of feather bud formation in the FFF region, molecular cues for axial morphogenesis of the wing along the 3D axes may be co-opted to modify morphogenesis of the feather bud, giving rise to flight feather-specific morphogenesis of traits. Such early modification would enable the feather bud to make at least some of the flight feather-specific traits, including direct connection with the bonny structure, quill knobs. Flight feathers in the wing connect with specific muscles for coordinating wing/feather movements and changing the wing to an airfoil shape [5], and the connection between the muscle and flight feather calamus, which is an important trait of the flight feather, is probably established during feather morphogenesis in the embryo.

Quill knobs are evident not only in extant bird species but also in basal taxa such as *Ichthyornis* and non-avian theropod dinosaurs such as *Velociraptor* [7]. Some non-avian theropod dinosaurs and basal taxa of birds are equipped with remex-type flight feathers with asymmetric shapes in their forelimb [45]. Analyses of juvenile specimens referable to the oviraptorosaur have suggested that morphological changes in remiges occurred during feather development [49]. Ancestors of birds might have already experienced the modification of early embryonic morphogenesis in the FFF region. This is a good example of how early modification of the embryonic morphogenetic process may have played a crucial role in the morphological evolution of key innovation.
